# Machine learning meets pK _a_

**DOI:** 10.12688/f1000research.22090.2

**Published:** 2020-04-27

**Authors:** Marcel Baltruschat, Paul Czodrowski

**Affiliations:** 1 Faculty of Chemistry and Chemical Biology, TU Dortmund University, Otto-Hahn-Strasse 6, 44227 Dortmund, Germany

**Keywords:** machine learning, pKa value, protonation, dissociation

## Abstract

We present a small molecule pK _a_ prediction tool entirely written in Python. It predicts the macroscopic pK _a_ value and is trained on a literature compilation of monoprotic compounds. Different machine learning models were tested and random forest performed best given a five-fold cross-validation (mean absolute error=0.682, root mean squared error=1.032, correlation coefficient r ^2^ =0.82). We test our model on two external validation sets, where our model performs comparable to Marvin and is better than a recently published open source model. Our Python tool and all data is freely available at https://github.com/czodrowskilab/Machine-learning-meets-pKa.

## Introduction

The acid-base dissociation constant (pK _a_) of a drug has a far-reaching influence on pharmacokinetics by altering the solubility, membrane permeability and protein binding affinity of the drug. Several publications summarize these findings in a very comprehensive manner ^1– 7^. An accurate estimation of pK _a_ values is therefore of utmost importance for successful drug design. Several (commercial and non-commercial) tools and approaches for small molecule pK _a_ prediction are available: MoKa ^8^ uses molecular interaction fields, whereas ACD/Labs Percepta Classic ^9^, Marvin ^10^ and Epik ^11^ make use of the Hammet-Taft equation. By means of Jaguar ^12^, a quantum-mechanical approach to pK _a_ prediction becomes possible. Recently, the usage of neural nets for pK _a_ prediction became prominent ^13– 15^. In particular, the publication by Williams *et al*. ^15^ makes use of a publicly available data set provided by the application DataWarrior ^16^ and provides a freely available pK _a_ prediction tool called OPERA.

As this article is part of a Python collection issue, we provide a pK _a_ prediction method entirely written in Python ^17^ and make it available open source (including all data). Our tool computes the macroscopic pK _a_ value for a monoprotic compound. Our model solely differentiates between a base and acid based on the predicted pK _a_ value; i.e., we do not offer separate models for acids and bases. In addition to pK _a_ data from DataWarrior ^16^, we also employ pK _a_ data from ChEMBL ^18^. As external validation sets, we use compound data provided by Novartis ^19^ and a manually curated data set compiled from literature ^20– 24^, which are not part of the training data.

## Methods

### Data set preparation

A ChEMBL ^18^ web search was performed to find all assays containing pK _a_ measurement data. The following restrictions were made: it must be a physicochemical assay, the measurements must be taken from scientific literature, the assay must be in “small-molecule physicochemical format” and the organism taxonomy must be set to “N/A”. This results in a list of 1140 ChEMBL assays downloaded as CSV file. Using a Python script, the CSV file was read in and processed further, extracting all additional information required from an internally hosted copy of the ChEMBL database via SQL. Only pK _a_ measurements, i.e. ChEMBL activities, were taken into account that were specified as exact (“standard_relation” equals “=”) and for which one of the following names was specified as “standard_type”: “pka”, “pka value”, “pka1”, “pka2”, “pka3” or “pka4” (case-insensitive). Measured values for which the molecular structure was not available were also sorted out. The resulting 8111 pK _a_ measured values were saved as SDF file.

A flat file from DataWarrior ^16^ named “pKaInWater.dwar” was used in addition. In this case, the file was converted to an SDF file only and contains 7911 entries with valid molecular structures.

These data sets were concatenated for the purpose of this study and preprocessed as follows:

- Removal of all salts from molecules
- Removal of molecules containing nitro groups, Boron, Selenium or Silicium
- Filtering by Lipinski‘s rule of five (one violation allowed)
- Keeping only pK _a_ data points between 2 and 12
- Tautomer standardization of all molecules
- Protonation of all molecules at pH 7.4
- Keeping only monoprotic molecules regarding the specified pK _a_ range
- Combination of data points from duplicated structures while removing outliers

All steps up to filtering out all pK _a_ values outside the range of 2 to 12 were performed with Python and RDKit ^25^. The QuacPac ^26^ Tautomers tool from OpenEye was used for tautomer standardization and setting the protonation state to pH 7.4. The Marvin ^10^ tool from ChemAxon was used to filter out the multiprotic compounds. It predicted the pK _a_ values of all molecules in the range 2 to 12 and then retained only those molecules where Marvin did not predict more than one pK _a_ in that range.

The removal of the outliers is performed in two steps. First, before combining multiple measurements for the same molecules, all entries where the pK _a_ predicted by ChemAxon's Tool Marvin differs from the experimental value by more than four log units are removed. All molecules were then combined on the basis of the canonical isomeric SMILES. In the second step, when combining several measured values of a molecule, all those values that deviate from the mean value by more than two standard deviations are removed. The remaining values are arithmetically averaged.

**Figure 1.**
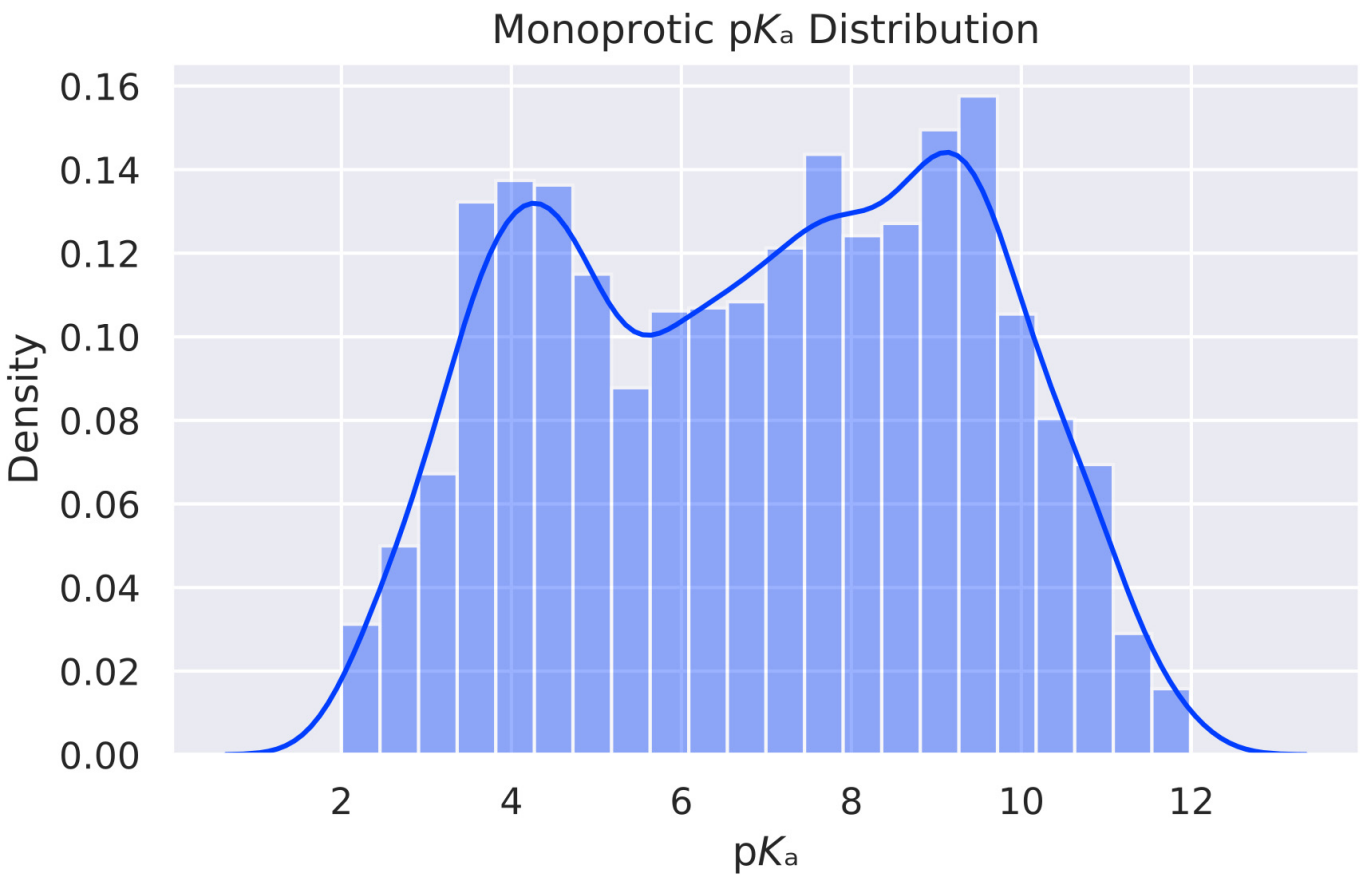
Distribution of the individual pK _a_ values.

**Figure 2.**
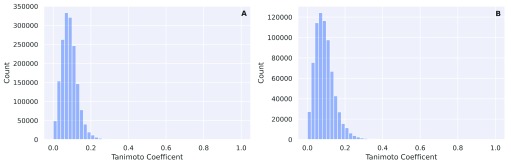
( **A**) Pairwise comparison between the training set and the Novartis test set (Fingerprint: 4096 bit MorganFeatures radius=3). ( **B**) Pairwise comparison between the training set and the test set compiled by manual curation (Fingerprint: 4096 bit MorganFeatures radius=3).

After all, 5994 unique monoprotic molecules with experimental pK _a_ values remained. The distribution of pK _a_ values is given in Figure 1. The same preprocessing steps were also performed on an external test data set provided to us by Novartis ^19^ (280 molecules) and a manual curation (123 molecules) from literature ^20– 24^. The Novartis data set consists of 280 unique molecules with a molecular weight between 129 and 670 daltons (mean value 348.68, standard deviation 94.17). The calculated LogP values vary between -1.54 and 6.30 (mean value 3.01, standard deviation 1.41). The 280 molecules spread over 228 unique Murcko Scaffolds. The ten most common murcko scaffolds cover 15% of the molecules of the total data set (42/280). A histogram of the pairwise comparison between the training set and the two external test sets (Fingerprint: 4096 bit MorganFeatures radius=3) is given in Figure 2(A) and Figure 2(B)

### Learning

First, to simplify cross-validation, a class “CVRegressor” was defined, which can serve as a wrapper for any regressor implementing the Scikit-Learn ^27^ interface. This class simplifies cross-validation itself, training and prediction with the cross-validated model. Next, 196 of the 200 available RDKit descriptors (“MaxPartialCharge”, “MinPartialCharge”, “MaxAbsPartialCharge” and “MinAbsPartialCharge” were not used because they are computed as “NaN” for many molecules), and a 4096-bit long MorganFeature fingerprint with radius 3 were calculated for the training data set. Random forest (RF), support vector regression (SVR, two configurations), multilayer perceptron (MLP, three configurations) and XGradientBoost (XGB) were used as basic regressors. Unless otherwise specified, the Scikit-Learn default parameters (version 0.22.1) were used. For the RF model, only the number of trees was increased to 1000. For SVR the size of the cache was increased to 4096 megabytes in the first configuration, but this only increases the training speed and has no influence on the model quality. In the second configuration the parameter “gamma” was additionally set to the value “auto”. For MLP in the first configuration the number of hidden layers was increased to two and the number of neurons per layer to 500. In the second configuration, early stopping was additionally activated, where 10% of the training data was separated as validation data. If the error of the validation data did not improve by more than 0.001 over ten training epochs, the training is stopped early to avoid overtraining. In the third configuration three hidden layers with a size of 250 neurons each were used with early stopping still activated. For XGB the default parameters of the used library XGBoost (version 0.90) ^28^ were applied. The training of RF, MLP and XGB was parallelized on 12 CPU cores and the generation of the folds for cross-validation as well as the training itself were random seeded to a value of 24 to ensure reproducibility. This resulted in a total of seven different machine learning configurations.

Six different descriptor/fingerprint combinations were also tested. First only the RDKit descriptors, followed by only the fingerprints and finally both combined. Additionally, all three combinations were tested again in a standardized form (z-transformed). As a result, 42 combinations of regressor and training data configurations were compared.

A 5-fold cross-validation was performed for all configurations, which were evaluated using the MAE, RMSE and the empirical coefficient of determination (r ^2^). After training was completed for all configurations, two external test data sets, which do not contain training data, were used to re-validate each trained cross-validated model. Here, MAE, RMSE, and r ^2^ were also calculated as statistical quality measures. To ensure that no training data was contained in the test data sets, the conical isomeric SMILES were checked for matches in both training and test data sets and corresponding hits were removed from the test data sets.

### Implementation

The following Python dependencies have to be met: Python >= 3.7, NumPy >= 1.18, Scikit-Learn >= 0.22, RDKit >= 2019.09.3, Pandas >= 0.25, XGBoost >= 0.90, JupyterLab >= 1.2, Matplotlib >= 3.1, Seaborn >= 0.9

For the data preparation pipeline, ChemAxon Marvin ^10^ and OpenEye QUACPAC/Tautomers ^26^ are required. To use the provided prediction model with the included Python script, ChemAxon Marvin ^10^ is not required.

First of all a working Miniconda/Anaconda installation is needed. Miniconda can be downloaded at https://conda.io/en/latest/miniconda.html.

Now an environment named "ml_pka" with all needed dependencies can be created and activated with:


conda env create -f environment.yml
conda activate ml_pka


Alternatively, a new environment can be created manually without the environment.yml file:


conda create -n ml_pka python=3.7
conda activate ml_pka


In case of Linux or macOS:


conda install -c defaults -c rdkit -c conda-forge scikit-learn rdkit xgboost jupyterlab matplotlib seaborn


In case of Windows:


conda install -c defaults -c rdkit scikit-learn rdkit jupyterlab matplotlib seaborn
pip install xgboost


### Operation

***Prediction pipeline.*** To use the data preparation pipeline the repository folder hast to be entered and the created conda environment must be activated. Additionally the Marvin ^10^ commandline tool cxcalc and the QUACPAC ^26^ commandline tool tautomers have to be added to the PATH variable.

Also the environment variables OE_LICENSE (containing the path to the OpenEye license file) and JAVA_HOME (referring to the Java installation folder, which is needed for cxcalc) have to be set.

After preparation a small usage information can be displayed with bash run_pipeline.sh -h. Examplary call:


bash run_pipeline.sh --train datasets/chembl25.sdf --test datasets/novartis_cleaned_mono_unique_notraindata.sdf


***Prediction tool.*** First of all the repository folder has to be entered and the created conda environment must be activated. To use the prediction tool the machine learning model has to be retrained. To do so the training script should be called, it will train the 5-fold cross-validated Random Forest machine learning model using 12 cpu cores. If the number of cores has to be adjusted the train_model.py can be edited by changing the value of the variable EST_JOBS.


python train_model.py


To use the prediction tool with the trained model QUACPAC/Tautomers have to be available as mentioned in the section above.

Now the python script can be called with an SDF file and an output path:


python predict_sdf.py my_test_file.sdf my_output_file.sdf


It should be noted that this model was built for monoprotic structures regarding a pH range of 2 to 12. If the model is used with multiprotic structures, the predicted values will probably not be correct.

## Results

### Different experimental methods

**Figure 3.**
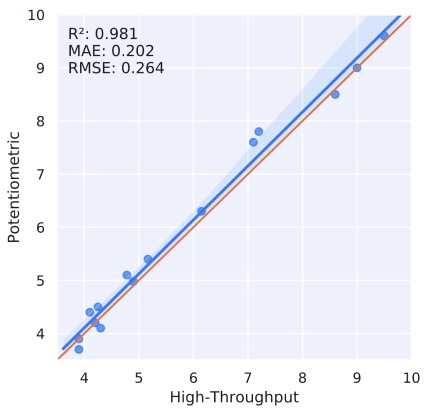
Correlation of Novartis compounds measured in potentiometric and high-throughput (capillary electrophoresis) set-up.

MAE, mean absolute error; RMSE, root mean square error.

One crucial point in the field of pK _a_ measurements (and its usage for pK _a_ predictions) was linked to the different experimental methods ^25, 30^. Based on the Novartis set, the correlation between capillary electrophoresis and potentiometric measurements (for 15 data points) was convincing enough (mean absolute error (MAE)=0.202, root mean squared error (RMSE)=0.264, correlation coefficient r ^2^=0.981) for us to combine pK _a_ measurements from these different experimental methods (see Figure 3).

**Figure 4.**
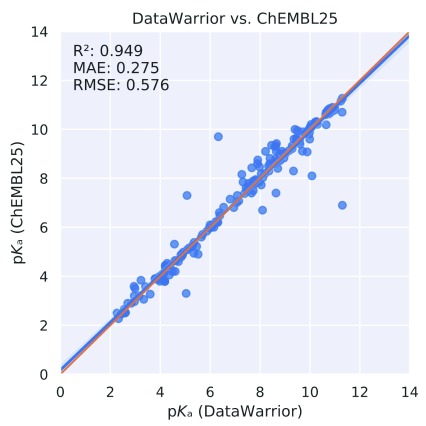
Intersection between ChEMBL and DataWarrior data sets.

MAE, mean absolute error; RMSE, root mean square error.

We also compared the pK _a_ values of 187 monoprotic molecules contained in both the ChEMBL and DataWarrior data sets. Due to the missing annotation, it remained unclear if different experimental methods were used or multiple measurements with the same experimental method have been performed (or a mixture of both). Either way, this comparison was an additional proof-of-concept that the ChEMBL and DataWarrior pK _a_ data sources can be combined after careful curation. The aforementioned intersection is given in Figure 4. The correlation coefficient between the annotated pK _a_ values for these two data sets r ^2^ was 0.949, the MAE was 0.275, and the RMSE was 0.576.

The compounds for which the pK _a_ values between the different sources deviate by more than two units are as follows:

- Hydralazine:
  - pK _a_ (DataWarrior) 5.075
  - pK _a_ (ChEMBL25) 7.3

- Edaravone:
  - pK _a_ (DataWarrior) 11.3
  - pK _a_ (ChEMBL25) 6.9

- Trifluoromethanesulfonamide:
  - pK _a_ (DataWarrior) 6.33
  - pK _a_ (ChEMBL25) 9.7

Since the annotation about the experimental settings is not given in the DataWarrior file, we can only hypothesize that these differences are due to the different experimental settings.

### Machine Learning

The statistics for a five-fold cross-validation are given in Table 1. In terms of the mean absolute error, a random forest with scaled MorganFeatures (radius=3) and descriptors gave the best performing model (MAE=0.682, RMSE=1.032, r ^2^=0.82). For the two external test sets (see Table 2), a random forest with FeatureMorgan (radius=3) gave the best model 

**Table 1.** Statistics of the five-fold cross-validation of the machine learning models. The two best and worst performing models are highlighted in green and red. For those neural networks where the values were specified as "not available" (“#NA”), the weights could not be optimized properly due to the large value range of the RDKit descriptors, so training failed here.

		Cross-Validation					
Modell (seed=24)	Train Configuration	MAE (mean)	MAE (std)	RMSE (mean)	RMSE (std)	R ^2^ (mean)	R ^2^ (std)
**Random Forest (n_estimators=1000)**	**Desc (196 RDKit)**	0,718	0,022	1,077	0,021	0,804	0,01
	**FCFP6 (4096 bit)**	0,708	0,021	1,094	0,029	0,797	0,008
	**Desc + FCFP6**	0,683	0,017	1,032	0,013	0,82	0,005
	**Desc (196 RDKit) (scaled)**	0,717	0,022	1,076	0,022	0,804	0,011
	**FCFP6 (4096 bit) (scaled)**	0,708	0,021	1,094	0,029	0,797	0,008
	**Desc + FCFP6 (scaled)**	0,682	0,017	1,032	0,013	0,82	0,005
**Support Vector Machine**	**Desc (196 RDKit)**	2,1	0,037	2,436	0,035	-0,004	0,004
	**FCFP6 (4096 bit)**	0,851	0,025	1,24	0,035	0,74	0,012
	**Desc + FCFP6**	2,1	0,037	2,436	0,035	-0,004	0,004
	**Desc (196 RDKit) (scaled)**	0,876	0,033	1,282	0,047	0,722	0,015
	**FCFP6 (4096 bit) (scaled)**	1,09	0,034	1,466	0,041	0,637	0,014
	**Desc + FCFP6 (scaled)**	1,02	0,037	1,4	0,047	0,668	0,016
**Support Vector Machine (gamma='auto')**	**Desc (196 RDKit)**	2,016	0,042	2,362	0,039	0,056	0,009
	**FCFP6 (4096 bit)**	1,612	0,031	1,926	0,033	0,373	0,007
	**Desc + FCFP6**	1,642	0,061	2,052	0,06	0,288	0,027
	**Desc (196 RDKit) (scaled)**	0,882	0,035	1,288	0,048	0,719	0,016
	**FCFP6 (4096 bit) (scaled)**	1,09	0,034	1,465	0,041	0,637	0,014
	**Desc + FCFP6 (scaled)**	1,019	0,037	1,4	0,047	0,669	0,016
**Multilayer Perceptron (hidden_layer_** **sizes=(500, 500))**	**Desc (196 RDKit)**	#NA	#NA	#NA	#NA	#NA	#NA
	**FCFP6 (4096 bit)**	0,866	0,025	1,27	0,047	0,727	0,019
	**Desc + FCFP6**	#NA	#NA	#NA	#NA	#NA	#NA
	**Desc (196 RDKit) (scaled)**	0,726	0,018	1,102	0,05	0,794	0,022
	**FCFP6 (4096 bit) (scaled)**	1,037	0,045	1,457	0,057	0,64	0,024
	**Desc + FCFP6 (scaled)**	0,968	0,032	1,383	0,04	0,677	0,014
**Multilayer Perceptron (hidden_layer_** **sizes=(500, 500),** **early_stopping=True)**	**Desc (196 RDKit)**	#NA	#NA	#NA	#NA	#NA	#NA
	**FCFP6 (4096 bit)**	0,894	0,024	1,297	0,04	0,715	0,016
	**Desc + FCFP6**	#NA	#NA	#NA	#NA	#NA	#NA
	**Desc (196 RDKit) (scaled)**	0,768	0,034	1,161	0,09	0,77	0,038
	**FCFP6 (4096 bit) (scaled)**	1,031	0,037	1,447	0,057	0,645	0,026
	**Desc + FCFP6 (scaled)**	0,984	0,029	1,404	0,035	0,666	0,017
**Multilayer Perceptron (hidden_layer_** **sizes=(250, 250, 250),** **early_stopping=True)**	**Desc (196 RDKit)**	#NA	#NA	#NA	#NA	#NA	#NA
	**FCFP6 (4096 bit)**	0,869	0,023	1,265	0,039	0,729	0,016
	**Desc + FCFP6**	#NA	#NA	#NA	#NA	#NA	#NA
	**Desc (196 RDKit) (scaled)**	0,775	0,008	1,158	0,033	0,773	0,013
	**FCFP6 (4096 bit) (scaled)**	1,026	0,038	1,455	0,053	0,642	0,022
	**Desc + FCFP6 (scaled)**	0,973	0,035	1,388	0,053	0,674	0,023
**XGBoost**	**Desc (196 RDKit)**	1,02	0,014	1,353	0,021	0,691	0,007
	**FCFP6 (4096 bit)**	1,094	0,027	1,423	0,036	0,657	0,011
	**Desc + FCFP6**	1,018	0,01	1,346	0,022	0,694	0,005
	**Desc (196 RDKit) (scaled)**	1,02	0,014	1,353	0,021	0,691	0,007
	**FCFP6 (4096 bit) (scaled)**	1,094	0,027	1,423	0,036	0,657	0,011
	**Desc + FCFP6 (scaled)**	1,018	0,01	1,346	0,022	0,694	0,005

MAE, mean absolute error; RMSE, root mean square error.

**Table 2.** Predictive performance of the machine learning models the on the two external test sets. The two best and worst performing models are highlighted in green and red. For those neural networks where the values were specified as "not available" (“#NA”), the weights could not be optimized properly due to the large value range of the RDKit descriptors, so training failed here.

		Novartis			AvLiLuMoVe		
Modell (seed=24)	Train Configuration	MAE	RMSE	R ^2^	MAE	RMSE	R ^2^
**Random Forest (n_estimators=1000)**	**Desc (196 RDKit)**	1,259	1,607	0,513	0,689	0,979	0,828
	**FCFP6 (4096 bit)**	1,147	1,513	0,569	0,532	0,785	0,889
	**Desc + FCFP6**	1,2	1,532	0,558	0,628	0,884	0,86
	**Desc (196 RDKit) (scaled)**	1,259	1,607	0,513	0,688	0,979	0,828
	**FCFP6 (4096 bit) (scaled)**	1,147	1,513	0,569	0,532	0,785	0,889
	**Desc + FCFP6 (scaled)**	1,198	1,531	0,558	0,628	0,884	0,86
**Support Vector Machine**	**Desc (196 RDKit)**	2,177	2,451	-0,132	2,18	2,441	-0,07
	**FCFP6 (4096 bit)**	1,423	1,732	0,435	0,688	0,981	0,827
	**Desc + FCFP6**	2,177	2,451	-0,132	2,18	2,441	-0,07
	**Desc (196 RDKit) (scaled)**	1,382	1,735	0,433	0,772	1,058	0,799
	**FCFP6 (4096 bit) (scaled)**	1,771	2,035	0,219	1,115	1,422	0,637
	**Desc + FCFP6 (scaled)**	1,746	2,015	0,235	1,044	1,345	0,675
**Support Vector Machine (gamma='auto')**	**Desc (196 RDKit)**	2,162	2,428	-0,111	1,921	2,242	0,097
	**FCFP6 (4096 bit)**	1,686	1,932	0,297	1,429	1,67	0,499
	**Desc + FCFP6**	2,161	2,442	-0,124	1,611	2,004	0,279
	**Desc (196 RDKit) (scaled)**	1,378	1,732	0,435	0,766	1,049	0,802
	**FCFP6 (4096 bit) (scaled)**	1,77	2,034	0,22	1,114	1,421	0,637
	**Desc + FCFP6 (scaled)**	1,744	2,013	0,236	1,043	1,343	0,676
**Multilayer Perceptron (hidden_layer_** **sizes=(500, 500))**	**Desc (196 RDKit)**	#NV	#NV	#NV	#NV	#NV	#NV
	**FCFP6 (4096 bit)**	1,414	1,773	0,407	0,852	1,169	0,755
	**Desc + FCFP6**	#NV	#NV	#NV	#NV	#NV	#NV
	**Desc (196 RDKit) (scaled)**	1,318	1,634	0,497	0,688	0,942	0,841
	**FCFP6 (4096 bit) (scaled)**	1,627	2,033	0,221	1,102	1,569	0,558
	**Desc + FCFP6 (scaled)**	1,542	1,941	0,29	1,001	1,427	0,634
**Multilayer Perceptron (hidden_layer_** **sizes=(500, 500), early_stopping=True)**	**Desc (196 RDKit)**	#NV	#NV	#NV	#NV	#NV	#NV
	**FCFP6 (4096 bit)**	1,404	1,772	0,408	0,846	1,154	0,761
	**Desc + FCFP6**	#NV	#NV	#NV	#NV	#NV	#NV
	**Desc (196 RDKit) (scaled)**	1,298	1,626	0,502	0,701	0,936	0,843
	**FCFP6 (4096 bit) (scaled)**	1,611	2,028	0,225	1,141	1,575	0,554
	**Desc + FCFP6 (scaled)**	1,605	1,998	0,248	0,987	1,365	0,665
**Multilayer Perceptron (hidden_layer_** **sizes=(250, 250, 250), early_stopping=True)**	**Desc (196 RDKit)**	#NV	#NV	#NV	#NV	#NV	#NV
	**FCFP6 (4096 bit)**	1,363	1,717	0,445	0,86	1,164	0,757
	**Desc + FCFP6**	#NV	#NV	#NV	#NV	#NV	#NV
	**Desc (196 RDKit) (scaled)**	1,354	1,705	0,452	0,777	1,057	0,799
	**FCFP6 (4096 bit) (scaled)**	1,584	1,989	0,254	1,053	1,468	0,613
	**Desc + FCFP6 (scaled)**	1,581	1,963	0,274	0,953	1,352	0,672
**XGBoost**	**Desc (196 RDKit)**	1,367	0,453	1,704	0,819	0,806	1,04
	**FCFP6 (4096 bit)**	1,28	0,503	1,624	0,782	0,823	0,992
	**Desc + FCFP6**	1,293	0,495	1,637	0,774	0,822	0,995
	**Desc (196 RDKit) (scaled)**	1,367	0,453	1,704	0,819	0,806	1,04
	**FCFP6 (4096 bit) (scaled)**	1,28	0,503	1,624	0,782	0,823	0,992
	**Desc + FCFP6 (scaled)**	1,293	0,495	1,637	0,774	0,822	0,995
**ChemAxon Marvin (V20.1.0)**		0,856	1,166	0,744	0,566	0,865	0,866
**OPERA (V2.5)** *		2,274	3,059	-0,754	1,737	2,182	0,124

*For OPERA 6 molecules from AvLiLuMoVe and 31 molecules from Novartis were left out because OPERA predicted either two or zero pK _a_ values. MAE, mean absolute error; RMSE, root mean square error.

- Novartis: MAE=1.147, RMSE=1.513, r ^2^=0.569
- LiteratureCompilation: MAE=0.532, RMSE=0.785, r ^2^=0.889)

The predictive performance for Marvin ^10^ and the OPERA tool ^15^ were as follows:

- Novartis
  - Marvin: MAE=0.856, RMSE=1.166, r ^2^=0.744
  - OPERA: MAE= 2.274, RMSE= 3.059, r ^2^= -0.754

- LiteratureComplation ^20– 24^
  - Marvin: MAE= 0.566, RMSE= 0.865, r ^2^= 0.866
  - OPERA: MAE= 1.737, RMSE= 2.182, r ^2^= 0.124.

This showed that our model had a slightly better performance than Marvin for the LiteratureCompilation, but Marvin performed better for the Novartis dataset. For both data sets, our models ^17^ had a better predictive performance than the OPERA tool. Since some molecules had to be omitted for prediction with OPERA due to none or multiple predicted pK _a_ values, no consistent significance test could be performed for all comparisons.

## Discussion and conclusions

The developed model offers the possibility to predict pK _a_ values for monoprotic molecules with good accuracy. However, since the model has been trained exclusively with monoprotic molecules, only monoprotic molecules can be predicted properly. In this respect the model is limited. Nevertheless, the results show that the performance for monoprotic molecules can compete with the performance of existing prediction tools. The good performance of Marvin on the Novartis set is interesting to note: the RMSE was almost 0.4 units better than our top performing model. This could be because Marvin’s training set is much larger than our own training set. This provides a better foundation for the training of the Marvin model. In contrast, Marvin performed slightly worse than our top model on the LiteratureCompilation. The OPERA tool performed significantly worse than our model on both external test sets. We assume that the addition of 2470 ChEMBL pK _a_ – datapoints to our training set which were not part of the OPERA training set led to this drop in predictive performance. In addition, the pre-processing of the data was performed differently by OPERA in comparison to our pre-processing procedure.

As next step for the enhancement and improvement of our pK _a_ prediction model ^17^, we are currently expanding it to multiprotic molecules. We are also investigating the impact of different neural net architectures and types (such as graph neural nets) and the development of individual models for acids and bases. From a chemistry perspective, an analysis of pK _a_ effects of different functional groups (e.g. by means of matched molecular pairs analysis) is an on-going effort for a future publication.

## Data availability

### Source data

Zenodo: czodrowskilab/Machine-learning-meets-pKa article. https://doi.org/10.5281/zenodo.3662245 ^17^.

The following data sets were used in this study:

- AvLiLuMoVe.sdf - Manually combined literature pK _a_ data.
- chembl25.sdf - Experimental pK _a_ data extracted from ChEMBL25.
- datawarrior.sdf - pK _a_ data shipped with DataWarrior.
- combined_training_datasets_unique.sdf - Preprocessed and combined data from datasets chembl25.sdf and datawarrior.sdf, used as training dataset.
- AvLiLuMoVe_cleaned_mono_unique_notraindata.sdf - used as external testset.
- novartis_cleaned_mono_unique_notraindata.sdf - inhouse dataset provided by Novartis ^19^, used as external testset.

The data sets are also available at https://github.com/czodrowskilab/Machine-learning-meets-pKa.

License: MIT license.

### Software availability

The source code is available at https://github.com/czodrowskilab/Machine-learning-meets-pKa.

Archived source code at time of publication: https://doi.org/10.5281/zenodo.3662245 ^17^.

License: MIT license.
