# Supplementary material for: Machine learning meets pK a
Source: F1000Res. 2020 Apr 27;9:Chem Inf Sci-113. Originally published 2020 Feb 13. [Version 2] doi: 10.12688/f1000research.22090.2 (PMC7096188; doi:10.12688/f1000research.22090.2)
Supplement: Peer review history

# Peer review: Reviewer response for version 1

**DOI:** 10.5256/f1000research.24362.r61511

**Published:** 2020-03-24

**Authors:** Johannes Kirchmair

**Affiliations:** 1 Department of Pharmaceutical Chemistry, Faculty of Life Sciences, University of Vienna, Vienna, Austria

Baltruschat and Czodrowski report on the development of a Python-based tool for the prediction of pk _a_ values. The tool is made available open source and will certainly be useful to the scientific community.

- Could the authors please comment on the structural relationship between the training and the test data (how far apart are these datasets, e.g. measured by the distribution of pairwise Tanimoto coefficients based on molecular fingerprints)?
- Could the authors please confirm statistical significance of the key statements made in the first paragraph of the Results section, starting with "This shows that our model slightly outcompetes..."?
- In the Abstract, please name the open source model that was tested as part of the comparative performance analysis.
- It is not clear from the manuscript text whether capillary electrophoresis and potentiometric measurements were the only two experimental methods considered in this work (or, for the Novartis dataset only).
- Please revise the sentence starting with "We also compare the overlap...".
- "make use of a" should be replaced by "makes use of a".
- Please explain where DataWarrior's data originates from.
- Please comment on the outliers observed in Figure 2.
- Please explain the procedure that was followed to ensure that there was no overlap between the training data and the external validation sets (the Novartis dataset in particular).
- "isomeric SMILES": is it canonical as well?
- Avoid using "you" in the Implementation section (use "one [can]..." instead).
- The part starting with "For the two external test sets..." is difficult to read. Could the authors find a more simple way to describe this and enable readers to compare the individual values more easily?

Is the work clearly and accurately presented and does it cite the current literature?

Yes

If applicable, is the statistical analysis and its interpretation appropriate?

Partly

Are all the source data underlying the results available to ensure full reproducibility?

Yes

Is the study design appropriate and is the work technically sound?

Yes

Are the conclusions drawn adequately supported by the results?

Yes

Are sufficient details of methods and analysis provided to allow replication by others?

Yes

Reviewer Expertise:

cheminformatics

I confirm that I have read this submission and believe that I have an appropriate level of expertise to confirm that it is of an acceptable scientific standard.

# Response

**Published:** 2020-04-15

**Authors:** Paul Czodrowski

**Affiliations:** TU Dortmund University, Germany

Could the authors please comment on the structural relationship between the training and the test data (how far apart are these datasets, e.g. measured by the distribution of pairwise Tanimoto coefficients based on molecular fingerprints)?

*We have included new figures (2A and 2B) with pairwise Tanimoto coefficients (4096 bit MorganFeatures radius=3).*

Could the authors please confirm statistical significance of the key statements made in the first paragraph of the Results section, starting with "This shows that our model slightly outcompetes..."?

*A thorough test of the statistical significance is hindered by the fact the external open source tool OPERA cannot process all molecules of our external test sets. We therefore decided to rephrase “This showed that our model slightly outcompeted Marvin for the LiteratureCompilation” to “his showed that our model has a slightly better performance than Marvin for the LiteratureCompilation”.*

In the Abstract, please name the open source model that was tested as part of the comparative performance analysis.

*The open source model is now mentioned in the abstract.*

It is not clear from the manuscript text whether capillary electrophoresis and potentiometric measurements were the only two experimental methods considered in this work (or, for the Novartis dataset only).

*We mention this already in the section “Different experimental methods” :“Due to the missing annotation, it remained unclear if different experimental methods were used or multiple measurements with the same experimental method have been performed (or a mixture of both)”.*

Please revise the sentence starting with "We also compare the overlap...".

*Changed "We also compared the overlap of the filtered (see next section) ChEMBL and DataWarrior data sets, 187 monoprotic molecules could be identified in both sources." To "We also compared the pKa values of 187 monoprotic molecules contained in both the ChEMBL and DataWarrior data *sets."

"make use of a" should be replaced by "makes use of a".

*Done*

Please explain where DataWarrior's data originates from.

*We cannot make any statement about the origin, because it is not included in the DataWarrior file.*

Please comment on the outliers observed in Figure 2.

*We include the name of the outliers and the different pKa values originating from different data sources, if they differ by more than two log units.*

Please explain the procedure that was followed to ensure that there was no overlap between the training data and the external validation sets (the Novartis dataset in particular).

*To ensure that no training data was contained in the test data sets, the canonical isomeric SMILES were checked for matches in both training and test data sets and corresponding hits were removed from the test data sets. Chirality was not included in this comparison.*

"isomeric SMILES": is it canonical as well?

*Yes, it is the canonical isomeric SMILES generated by RDKit.*

Avoid using "you" in the Implementation section (use "one [can]..." instead).

*Done*

The part starting with "For the two external test sets..." is difficult to read. Could the authors find a more simple way to describe this and enable readers to compare the individual values more easily?

*We have pulled most of the numbers apart and included bullet points which improves the readability and makes a comparison more feasible.*

# Peer review: Reviewer response for version 1

**DOI:** 10.5256/f1000research.24362.r60011

**Published:** 2020-02-25

**Authors:** Ruth Brenk

**Affiliations:** 1 Department of Biomedicine, University of Bergen, Bergen, Norway

The article describes a machine learning method for pkA prediction. The presented method and results are scientifically solid. Therefore, I recommend the article for indexing. However, the methods and results should be presented more clearly before the article is indexed.

 The following points should be addressed:

1. Past tense should be used consistently to describe the methods and results.
2. The “different experimental methods” should be moved to the results part as it contains results.
3. A short description of the molecules in the self-compiled data set and the Novartis set should be added. What type of molecules are contained in these sets?
4. Why does the user need to train the model before making predictions? Could the trained model be provided to make the developed tool easier to use?
5. In the discussion section, the developed method should be first discussed before discussing its performance relative to other methods.

Is the work clearly and accurately presented and does it cite the current literature?

Partly

If applicable, is the statistical analysis and its interpretation appropriate?

Not applicable

Are all the source data underlying the results available to ensure full reproducibility?

Yes

Is the study design appropriate and is the work technically sound?

Yes

Are the conclusions drawn adequately supported by the results?

Yes

Are sufficient details of methods and analysis provided to allow replication by others?

Yes

Reviewer Expertise:

chemoinformatics

I confirm that I have read this submission and believe that I have an appropriate level of expertise to confirm that it is of an acceptable scientific standard.

# Response

**Published:** 2020-03-05

**Authors:** Paul Czodrowski

**Affiliations:** TU Dortmund University, Germany

1. Past tense should be used consistently to describe the methods and results. *We have used past tense throughout the entire document.*
2. The “different experimental methods” should be moved to the results part as it contains results. *This part was moved accordingly.*
3. A short description of the molecules in the self-compiled data set and the Novartis set should be added. What type of molecules are contained in these sets? *We have added this information in the manuscript: "The Novartis data set consists of 280 unique molecules with a molecular weight between 129 and 670 daltons (mean value 348.68, standard deviation 94.17). The calculated LogP values vary between -1.54 and 6.30 (mean value 3.01, standard deviation 1.41). The 280 molecules spread over 228 unique Murcko Scaffolds. The ten most common murcko scaffolds cover 15% of the molecules of the total data set (42/280)."*
4. Why does the user need to train the model before making predictions? Could the trained model be provided to make the developed tool easier to use? *The user has to train the model again by himself or herself, because the model is larger than 1.7 GB as a file and therefore cannot be offered for download without paid services. Even with the best possible bzip2 compression, the file is still over 250 MB in size and thus larger than the 100 MB limit at GitHub. In addition, if the model is provided as a file, you must use exactly the same versions of the Python libraries used for training to load the model, otherwise inconsistencies may occur. However, if the model is trained again by yourself, only the minimum library requirements have to be met.*
5. In the discussion section, the developed method should be first discussed before discussing its performance relative to other methods. *This was done and is reflected in the new manuscript version.*
